# Urethral Bulking Agents for the Treatment of Urinary Incontinence: Efficacy, Safety, and Impact on the Overactive Bladder Symptoms with an Underlying Detrusor Overactivity

**DOI:** 10.3390/jcm13195810

**Published:** 2024-09-28

**Authors:** Maurizio Serati, Maria Rosaria Campitiello, Marco Torella, Giada Mesiano, Chiara Scancarello, Fabio Ghezzi, Andrea Papadia, Elena Gamarra, Giorgio Caccia, Andrea Braga

**Affiliations:** 1 Department of Obstetrics and Gynecology, Del Ponte Hospital, University of Insubria, 21100 Varese, Italy; giadamesiano01@gmail.com (G.M.); chiara.scancarello@asst-settelaghi.it (C.S.); fabio.ghezzi@uninsubria.it (F.G.); 2 Department of Obstetrics and Gynecology and Physiopathology of Human Reproduction, ASL Salerno, 84131 Salerno, Italy; rosycampitiello@gmail.com; 3 Department of Gyanecology, Obstetric and Reproductive Science, Second University of Naples, 80138 Naples, Italy; marcotorella@iol.it; 4 Department of Obstetrics and Gynecology, Ente Ospedaliero Cantonale—Civico Hospital, 6900 Lugano, Switzerland; andrea.papadia@eoc.ch; 5 Faculty of Biomedical Sciences, Università della Svizzera Italiana, 6900 Lugano, Switzerland; andrea.braga@eoc.ch; 6 Department of Endocrinology and Diabetology, EOC—Civico Hospital, 6900 Lugano, Switzerland; elena.gamarra@eoc.ch; 7 Department of Obstetrics and Gynecology, EOC—Beata Vergine Hospital, 6850 Mendrisio, Switzerland; giorgio.caccia@eoc.ch

**Keywords:** mixed urinary incontinence, urethral bulking agents, stress urinary incontinence, urge urinary incontinence, overactive bladder syndrome, macroplastique

## Abstract

**Background**: Mixed urinary incontinence (MUI) has always represented a major therapeutic challenge and the management of this type of incontinence is often complicated by uncertain outcomes. Surgical options include interventions targeting both stress urinary incontinence (SUI) and urge urinary incontinence (UUI), although there are no international published guidelines that dictate whether it is better to start with surgical management to address the SUI or UUI component after the failure of conservative treatment. The aim of the present study is to evaluate the effectiveness of the Macroplastique (MPQ) procedure on overactive bladder (OAB) symptoms in women with MUI with a minimum follow-up of 1 year. **Methods**: A retrospective analysis of prospectively collected data was performed in two tertiary reference centers. We enrolled all women complaining of symptoms of SUI and OAB, dry or wet, with a urodynamically confirmed diagnosis of MUI [urodynamic stress incontinence (USI) with detrusor overactivity (DO)], who took a previous ineffective antimuscarinic treatment and underwent the MPQ procedure. We considered as objectively cured women who did not leak urine during the stress test and with a 1 h pad-test negative, while International Consultation on Incontinence Questionnaire–Short Form (ICIQ-SF), ICIQ-OAB, Patient Global Impression of Improvement (PGI-I) scale, and a Visual Analogue Scale (VAS) were used to assess subjective outcomes. **Results**: A total of forty-six patients who met the inclusion criteria and who underwent the MPQ procedure were considered for the analysis. At the 1-year mark of follow-up, 72% of patients were objectively cured at stress test and 65% were objectively cured at pad-test, while 72% of women declared themselves subjectively cured. OAB symptoms significantly improved after MPQ and a complete resolution of OAB was recorded in 35% of patients. **Conclusions**: This study demonstrated that MPQ is a safe and effective option for the treatment of female MUI. Furthermore, MPQ significantly improves the symptoms of OAB and is able to completely cure this condition in a relevant percentage of women with MUI when pharmacological treatment fails.

## 1. Introduction

MUI, defined as the involuntary loss of urine associated with the sensation of urgency and also associated with exertion, effort, sneezing, or coughing [1], has always represented a major therapeutic challenge. The management of this type of incontinence is often complicated by uncertain outcomes, as patients may present with more severe symptoms and a worse quality of life (QoL) than patients who complain of pure symptoms of SUI or UUI. The prevalence of MUI accounts for 35% of incontinence observed in daily clinical practice [2], however it varies widely (ranges from 8.3% to 93.3% [3]) depending on the definition used. Women of all ages are affected, but aging is associated with a steadily increasing risk of MUI.

In women with MUI, the dilemma is always the same: which component to treat first? Treatment should be focused on the symptom that has the greatest negative impact on QoL. Nevertheless, patients are often unable to establish which component is more troublesome. For this reason, appropriate counseling of the patient with regard to their expectations and therapeutic goals is mandatory [4].

Leading international urological and urogynecological associations propose the surgical management of MUI after failure of conservative and pharmacological treatment.

Surgical options include interventions targeting both SUI and UUI, although there are no internationally published guidelines that dictate whether it is better to start with surgical management to address the SUI or the UUI component [5]. In case of stress-predominant MUI (SP-MUI), it is usually recommended to start with a less invasive procedure; however, mid-urethral slings (MUS) are the most widely used procedures in contemporary practice. A systematic review and meta-analysis of MUS in women with MUI [6] reported an overall subjective cure rate of 56.4% among women with a mean follow up of approximately 34.9 months. In this study, which included five randomized controlled trials (RCT) and seven prospective studies, the stress component cure rate was high at 85–97%, while the urge component cure rate was more variable, ranging from 30 to 85%. In a follow-up study of 1113 women with MUI [7] treated with tension-free vaginal tape trans-obturator (TVT-O) and analyzed according to the women’s predominant bother: SUI, UUI, or both incontinence equally, authors reported that SUI was cured equally in SP-MUI and urgency-predominant MUI (UP-MUI). However, women with SP-MUI had significantly better overall cure rate at both 7 and 38 months than women with UP-MUI. Recently, a small case series of studies, found that UUI symptoms improve in 30–85% of patients with MUI undergoing MUS procedures [8]. Nevertheless, the durability of this improvement may be limited. Zyczynski et al. [9], in a secondary analysis of data from three multicenter urinary incontinence surgical trials of women with SP-MUI assigned to Burch colposuspension, autologous fascial sling, retropubic or trans-obturator MUS, showed that the proportion of women who reported ≥70% improvement in UUI, declined from 50 to 64.3% at 1 year to 36.5 to 54.1% at the 5-year (*p* < 0.001) follow-up. In another large prospective cohort study [10] that included 754 consecutive women with SUI and urgency and 514 women with SUI and UUI undergoing MUS, the authors reported an overall rate of persistent symptoms of urgency and UUI after MUS implantation of 40.3% and 32.3%, respectively. Preoperative DO was found as independent risk factor for the continued presence of urgency and UUI symptoms [11].

Although the use of MUS for the treatment of MUI appears to be a viable option [12,13], after the US Food and Drug Administration (FDA) warning in 2019 [14], this type of surgery has been widely debated in recent years due to a lack of long-term data and the rate of mesh erosion. Furthermore, persistent urinary urgency after MUS treatment is prevalent and ranges from 30 to 70% [3].

For these reasons, suitable alternatives to MUS are being considered. Recent data on the use of urethral bulking agents (UBA) appear to be very promising. Several studies have demonstrated that UBA, particularly Macroplastique (MPQ), are significantly associated with a lower intra- and postoperative complication rate than MUS, showing a similar subjective satisfaction rate [15,16]. In addition, a similar improvement in sexual function was reported, but with a lower rate of de novo dyspareunia [17,18].

However, a few studies in the literature have evaluated the effectiveness of UBA in patients with MUI, focusing in particular on the assessment of the stress component, but none have evaluated the effects of UBA on the urgency component. Most of these studies reported heterogeneous results from non-standardized study designs that did not allow for a clear interpretation of the outcomes [19].

The aim of the present study was to evaluate the effectiveness of the MPQ procedure on OAB symptoms in women with MUI with a minimum follow-up of 1 year.

## 2. Materials and Methods

This is a retrospective analysis of prospectively collected data performed in two tertiary reference centers. We considered all women who came to our units from January 2019 and June 2023 complaining of symptoms of SUI and OAB, dry or wet, with a urodynamically confirmed diagnosis of MUI (USI with DO), who took a previous ineffective antimuscarinic treatment and underwent the MPQ procedure.

Exclusion criteria were women with concomitant POP greater than stage 1 according to the POP quantification system [20], voiding dysfunction (postvoid residual > 100 mL evaluated by ultrasound) and women with history of radical pelvic surgery or psychiatric and neurologic disorders.

Preoperative evaluation included medical history, urinalysis, a 3-day bladder diary and physical examination with POP-Q system evaluation. The urodynamic study (UDS), including uroflowmetry, filling cystometry, Valsalva leak-point pressure (VLPP) measurement, and pressure/flow study, was performed by trained urogynecologists according to the urodynamic practice guidelines of the International Continence Society [21]. Urethral hypermobility was referred to as Q-tip test > 30°. Women were included independently of Q-tip and VLPP test values.

Each patient completed the ICIQ-SF and ICIQ-OAB at baseline and at a 1-year follow-up. Surgical procedures were performed under general or spinal anesthesia (depending on anesthesiologic requirements and/or patient preference), by expert surgeons.

Post-operative assessment, mandatory at 1 year after surgery, included clinical history, physical examination, bladder diary, orthostatic cough stress test with bladder filling greater than 400 mL (measured by ultrasound), 1 h pad-test and subjective satisfaction assessment.

The ICIQ-SF, ICIQ-OAB, PGI-I scales, and two VAS scales (one to evaluate the outcomes of SUI symptoms and one for the outcomes of OAB symptoms) with individual response from 0 to 10 to evaluate the degree of patient satisfaction with continence (with 0 representing “not satisfied” and 10 “satisfied”) were used to evaluate subjective outcomes [18]. The ICIQ-UI Short Form is a questionnaire for evaluating the frequency, severity, and impact on quality of life (QoL) of urinary incontinence in men and women in research and clinical practice across the world. This short and simple questionnaire is also of use to general practitioners and clinicians in both primary and secondary care institutions to screen for incontinence, to obtain a brief yet comprehensive summary of the level, impact and perceived cause of symptoms of incontinence, and to facilitate patient-clinician discussions. The number of items is 4 and the scoring scale is 0–21. The ICIQ-OAB is a questionnaire for evaluating overactive bladder and related impact on quality of life (QoL) and outcome of treatment in men and women in research and clinical practice across the world. The ICIQ-OAB provides a measure to assess the impact of urinary frequency, urgency, urge incontinence, and nocturia symptoms. The number of items is 4 and the scoring scale is 0–16. The PGI-I (Patient Global Impression of Improvement) scale is a 7-point scale, with a range of responses from 1, “very much improved”, through 7, “very much worse”.

We considered objectively cured, in terms of the SUI component, women who did not present urine leakage during the stress test and with a negative 1 h pad test. Women were subjectively considered cured by the MPQ procedure when the PGI-I score was ≤2 and the SUI VAS score ≥ 8 [12,13,18].

We considered women improved, in terms of OAB symptoms, in case of reduction in ICIQ-OAB scores after UBA and cured in case of postoperative ICIQ-OAB score ≤ 4 and OAB vas score ≥ 8.

The Declaration of Helsinki was followed, and preoperative written informed consent was obtained from all patients in this observational prospective evaluation. The study did not require Ethical/Institutional review board approval because we retrospectively analyzed the patients and normal clinical practice was followed.

## 3. Statistical Analysis

IBM-SPSS v. 17 for Windows (IBM Corp, Armonk, NY, USA) was used to perform statistical analysis. Continuous variables were reported as median and interquartile range. We used the χ^2^ test to analyze and compare the surgical outcomes during the follow-up. One-way analysis of variance was used to compare continuous series of variables in the comparison of the scores used to measure the subjective outcomes.

## 4. Results

Forty-six patients who met the inclusion criteria and who underwent the MPQ procedure were considered for the analysis. Table 1 reports the baseline characteristics of the patients. The median age of the study group was 53 (45.5–65.5). Preoperative urodynamic data were reported in Table 2. During the study period, no patients were lost to follow-up and all women completed the evaluation at 1 year.

Data regarding objective cure rates are summarized in Table 3a, while Table 3b shows the score changes from baseline to 1-year follow-up of ICIQ-SF and ICIQ-OAB and the subjective outcomes according to PGI-I score, in particular the rate of women responding “very much better” or “much better” on this scale.

At the 1-year follow-up, 33 out of 46 patients (72%) were objectively cured at stress test and 30 out 46 patients (65%) were objectively cured at pad-test, while seventy-two percent of women (33 out 46) declared themselves subjectively cured

Furthermore, we found a statistically significant improvement of both ICIQ-SF score (14 versus 4; *p* < 0.0001) and ICIQ-OAB score (15 versus 5.5; *p* < 0.0001) from the baseline to 1 year after MPQ procedure.

Overall OAB improvement rate was found in 20 out 46 patients (43.5%); in 16 of them (16 out 46, 35%), a complete resolution of OAB symptoms was recorded. Among the other patients, 7 required a further therapy for OAB, such as Botox or combination therapy (antimuscarinic plus beta-adrenergic). Of these, 4 women had resolution of OAB symptoms.

Table 4 shows the Clavien-Dindo classification of 1-year complications of MPQ.

We recorded 3 women (6.5%) with persistence, or de novo onset of urinary tract infections (UTI) treated with antibiotic prophylaxis, while only one woman (2%) reported dyspareunia treated with local estrogen therapy.

## 5. Discussion

This retrospective analysis of prospectively collected data reports the outcomes of OAB symptom evolution in women undergoing MPQ procedure for the treatment of female MUI with urodynamically confirmed diagnosis of SUI with DO.

To the best of our knowledge, there are not studies focused on this issue available in the literature so far. We have shown that MPQ is an effective procedure on OAB symptoms in a proportion of women with MUI whose pharmacological treatment fails.

We found that more than one-third of this population achieved total resolution of OAB symptoms and significant improvement in these symptoms in the overall study population. This is why only 15% of women required a further treatment for OAB one year after surgery. In addition, we found a high overall objective (72%) and subjective (72%) cure rate of MUI symptoms.

MUI is a very common condition, although its real prevalence is not well known due to different definitions used. Brubaker et al., in two similar studies, which enrolled women who underwent surgery for SUI and non-surgical treatment for UUI, showed that the prevalence of MUI assessed by validated questionnaires ranged from 8% to 93% and from 64% to 96%, respectively [3,22]. Moreover, patients with MUI often present more severe symptoms, making their treatment very difficult and consequently less effective.

Managing patient expectations is crucial, especially in women undergoing surgery for SUI. In fact, these patients often believe that the urge component will also disappear after surgery. As demonstrated by several studies, persistent urgency is the most common reason for dissatisfaction after MUS [23]. The problem is that underlying causes of OAB are not well understood but are believed to be multifactorial in nature. The etiology may be related to bladder hypersensitivity, low bladder compliance, or DO [24,25]. The latter, defined by the IUGA/ICS [1] as “the occurrence of involuntary detrusor contractions during filling cystometry, which may be spontaneous or provoked”, leads to OAB (wet or dry) in about 90% of cases, with no recognizable etiology.

First-line treatment for OAB includes behavioral therapy, such as reducing weight and fluid intake, bladder training, and pelvic floor muscle training, as well as pharmacological treatment with antimuscarinic drugs and β3-adrenergic agonists [25]. Patients with refractory OAB (rOAB) may have more severe symptoms or underlying pathophysiology that were not resolved by the first line medication [24]. Furthermore, it can significantly impair women’s QoL. However, there is no consensus on the definition of rOAB, and often it is based on a physician’s subjective judgment. As highlighted by Peyronnet et al. [26] in a comprehensive review of OAB pathophysiology, the identification of the mechanisms on the basis of OAB symptoms may help to tailor treatment to individual patients and improve outcomes. OAB and, in particular, in the case of rOAB, could be sub-grouped into different phenotypes based on different clinical presentations and treatment results [24]. In our study, we focused on the urethrogenic hypothesis: urgency can be caused by the activation of urethral afferents by urethral perfusion that can modulate the micturition reflex [27]. The hypothesis is that the entry of urine into the proximal urethra in patients with SUI may stimulate urethral afferents, inducing and/or increasing DO [27].

It is well recognized that SUI surgery improves lower urinary tract symptoms (LUTS) in patients with MUI, but the role of SUI surgery for isolated OAB (particularly dry OAB) in selected patients with “urethral urgency” has not yet been assessed [26]. Moreover, pharmacological treatment seems to be less effective. In our previously published study [28], we analyzed the efficacy of solifenacin in patients with de novo OAB after TVT-O, hypothesizing that OAB may be caused by a weak urethral sphincter mechanism, resulting in funneling of the proximal urethra. We prospectively compared 110 women with de novo OAB (group 1) at 3 months after placement of TVT-O, with 120 consecutive naïve patients with OAB symptoms without a previous surgical procedure for SUI (group 2), prescribing a 12-week antimuscarinic therapy with oral solifenacin 5 mg once daily. We found that group 1 had significantly less benefit in the mean decrease in urgency and UUI episodes daily (−1.1 vs. −2.3 and −0.2 vs. −1.1, respectively, each *p* < 0.0001). In group 1 we also found a lower subjective solifenacin effect. 

Another systematic review of the literature and meta-analysis [29] on de novo OAB following MUS procedures showed that the overall incidence of de novo OAB following Midurethral sling procedures is approximately 9%.

On the contrary, in terms of risk of postoperative OAB symptoms, the available data on the use of UBA appear to be very promising [30,31,32]. There is some evidence to support that by addressing urethral insufficiency with an anti-incontinence procedure, it is possible to improve urgency symptoms in patients with stress-induced DO [33,34]. On the basis of these results, we assumed that UBA may support the laxity of the urethra specifically in proximity to the bladder neck, reducing the funneling and preventing the activation of urethral afferents due to urine entering the proximal urethra.

In fact, in our study, we have shown that MPQ is an effective option for female MUI, not only for the SUI component but also for rOAB after pharmacological treatment failure.

Points of strength of the present study include (1) that the study population was extremely homogeneous, with strict exclusion and inclusion criteria; (2) the use of validated tools for the assessment of subjective and objective outcomes; (3) no patients were lost to follow-up at either study center.

Conversely, the limitations of this study could include (1) retrospective design, (2) limited sample size, (3) lack of data regarding quality-of-life (QoL) (but there is no validated QoL questionnaire in Italian), about the timing of the onset of OAB symptoms and of urodynamic data after the bulking agent procedures, and(4) inability to distinguish subjective improvement results between SUI and OAB.

## 6. Conclusions

The results of this study demonstrated that MPQ is a safe and effective option for the treatment of female MUI. Furthermore, MPQ significantly improves the symptoms of OAB and is able to completely cure this condition in a relevant percentage of women with MUI, when pharmacological treatment fails.

## Figures and Tables

**Table 1:** Baseline patient’s characteristics.

Characteristics	N = 46
Age, yr, median, (IQR)	53 (45.5–65.5)
BMI, kg/m^2^, median, (IQR)	27 (24–31)
Menopausal, no. (%)	29 (63)
HRT, no. (%)	2 (7)
Recurrent UTI, no. (%)	3 (6.5)
Previous vaginal deliveries, median, (IQR)	2 (2–3)
Macrosome (≥4000 g), no. (%)	6 (13)
Operative delivery (vacuum/forceps), no. (%)	1 (2)
Smoking habits, no. (%)	3 (6.5)

IQR: interquartile range; BMI: body mass index; HRT: hormone replacement therapy; UTI: urinary tract infection.

**Table 2:** Preoperative urodynamic data.

Urodynamic Parameters	N = 46
FDTV (mL)	160 (50–200)
CC (mL)	410 (320–500)
PDetMax during filling phase (cmH_2_O)	18 (12–20)
Qmax (mL/s)	28.5 (17–36.5)
PDetMax during voiding (cmH_2_O)	31.5 (10–75)
PDetQMax (cmH_2_O)	35 (25.5–69)
VLPP (cmH_2_O)	64 (24–91)

Data are expressed as median (interquartile range); FDTV = First Desire to Void; CC = Cystometric Capacity. PDetMax = Maximum Detrusor Pressure; Qmax = Maximum Flow; PDetQMax = Detrusor Pressure at Maximum Flow; VLPP = Valsalva Leak Point Pressure.

**Table 3:** Cure rates at 1 year follow-up visit.

**a. Objective Cure Rates SUI.**			
		1 year	
Objectively cured Objectively cured (at stress test)		72% (33/46)	
Objectively cured (at pad test)		65% (30/46)	
**b. Subjective outcomes scores**.			
	baseline	1 year	*p* value
ICIQ-sf	14 (12–17)	4 (3–9)	<0.0001 *
“Very much better” or “much better” on PGI-I		33/46 (72%)	
ICIQ-OAB	15 (15–16)	5.5 (4–10)	<0.0001 *

Data are expressed as median (interquartile range); * One-way analysis of variance (ANOVA).

**Table 4:** Clavien-Dindo Classification of 1-yr complications.

Complications	N = 46	Action
CLAVIEN II		
Persistence or de novo onset of UTIs	3/46 (6.5%)	Antibiotic prophylaxis
de novo dyspareunia, no. (%)	1/46 (2%)	Local estrogenic therapy

## Data Availability

The data cannot be published for privacy reasons. The database may be available to the editor.
